# Nanoscale morphology, optical dynamics and gas sensor of porous silicon

**DOI:** 10.1038/s41598-024-54336-x

**Published:** 2024-02-14

**Authors:** Atefeh Ghaderi, Jamshid Sabbaghzadeh, Laya Dejam, Ghobad Behzadi Pour, Emad Moghimi, Robert S. Matos, Henrique Duarte da Fonseca Filho, Ștefan Țălu, Amirhossein Salehi shayegan, Leila Fekri Aval, Mahdi Astani Doudaran, Amirhossein Sari, Shahram Solaymani

**Affiliations:** 1 Quantum Technologies Research Center (QTRC), Science and Research Branch, Islamic Azad University, Tehran, Iran; 2 Department of Physics, Islamic Azad University, West Tehran Branch, Tehran, Iran; 3 Department of Physics, East Tehran Branch, Islamic Azad University, Tehran, 18661-13118 Iran; 4 Faculty of Physics, Kharazmi University, Tehran, Iran; 5 Amazonian Materials Group, Physics Department, Federal University of Amapá-UNIFAP, Macapá, Amapá Brazil; 6 Laboratory of Synthesis of Nanomaterials and Nanoscopy, Physics Department, Federal University of Amazonas-UFAM, Manaus, Amazonas Brazil; 7 The Directorate of Research, Development and Innovation Management (DMCDI), The Technical University of Cluj-Napoca, Constantin Daicoviciu Street, No. 15, Cluj-Napoca, 400020 Cluj County, Romania

**Keywords:** Gas sensor performance, Morphological properties, Optical properties, Porous silicon, Thin films, Scanning probe microscopy, Surfaces, interfaces and thin films, Sensors and biosensors

## Abstract

We investigated the multifaceted gas sensing properties of porous silicon thin films electrodeposited onto (100) oriented P-type silicon wafers substrates. Our investigation delves into morphological, optical properties, and sensing capabilities, aiming to optimize their use as efficient gas sensors. Morphological analysis revealed the development of unique surfaces with distinct characteristics compared to untreated sample, yielding substantially rougher yet flat surfaces, corroborated by Minkowski Functionals analysis. Fractal mathematics exploration emphasized that despite increased roughness, HF/ethanol-treated surfaces exhibit flatter attributes compared to untreated Si sample. Optical approaches established a correlation between increased porosity and elevated localized states and defects, influencing the Urbach energy value. This contributed to a reduction in steepness values, attributed to heightened dislocations and structural disturbances, while the transconductance parameter decreases. Simultaneously, porosity enhances the strength of electron‒phonon interaction. The porous silicon thin films were further tested as effective gas sensors for CO_2_ and O_2_ vapors at room temperature, displaying notable changes in electrical resistance with varying concentrations. These findings bring a comprehensive exploration of some important characteristics of porous silicon surfaces and established their potential for advanced industrial applications.

## Introduction

Silicon has undergone extensive research, and its technological applications have reached advanced levels, establishing it as a widely utilized material in diverse scientific fields beyond electronics. Porous silicon is a material that can be obtained through the chemical or electrochemical dissolution of crystalline silicon, first fabricated by Uhlir^1^. It has been extensively studied for its mechanical, electrical, and optical properties, as well as its potential applications in sensing and optoelectronics^2–5^. Various methods of fabrication have been reported^6,7^, with electrochemical etching being one of the most common^8^. Porous silicon offers advantages such as low-temperature performance, low cost, easy manufacturing, and compatibility with silicon technology in electronics, as well as a high surface-to-volume ratio, making it useful in different industries^9^.

The investigation of the surface characteristics of a porous silicon system is a crucial aspect guiding its technological applications. Our study delves into the behavior of spatial patterns, shedding light on how they can impact the material's physical and optical properties. In the realm of surface morphology studies, researchers traditionally rely on techniques like scanning electron microscopy (SEM) or atomic force microscopy (AFM). However, AFM stands out as a precision tool for comprehensive assessment of a surface's topographical properties^10,11^. Due to its sensitivity and accuracy, AFM generates 3D topographic maps, yielding diverse morphological parameters and Minkowski functionals (MFs)^12,13^. These insights prove invaluable for characterizing surfaces across various scales. Statistical analysis of AFM images involves extracting quantitative height-based parameters like roughness root mean square surface roughness (Sq), average roughness (Sa), maximum peak height (Sp), maximum pit depth (Sv), and maximum height (Sz), and more. These metrics quantitatively define surface texture, enabling the identification of variations, irregularities, and patterns^14,15^. Additionally, AFM allows for the study of 3D spatial patterns through fractal mathematics, shedding light on specific aspects of sample microtexture^16–18^. Herein, we have incorporated new parameters, such as fractal succolarity (FS) and topographic entropy (E), to elucidate surface porosity and the uniformity of 3D topographic profiles in our samples. These aspects are crucial as nanoscale exploration of the porous silicon surface plays a pivotal role in unraveling intricate surface features, understanding optical behaviors, and optimizing potential applications.

The reflectance spectra of porous silicon have been analyzed using a simulation method that adjusts dielectric function models. Silicon thin films are utilized in devices for optics, optoelectronics, and microelectronics due to its band gap of 1.12 eV, which enables efficient detection of visible‒light and conversion of sunlight into electricity^19^. The potential technological importance of silicon-based light emitting devices has sparked interest in the visible photoluminescence of porous silicon at room temperature, which was first reported by Canham in 1990^20^ and subsequently confirmed by other researchers^21^. However, the existing literature on porous silicon system reveals ongoing controversies regarding its optical and morphological properties under varying preparation conditions, particularly in electrodeposition systems, so far.

Remarkably, in recent years, gas sensing has become increasingly important in environmental monitoring, with a focus on developing small size, low‒power consumption, and reliable gas sensors. Different sensor configurations are required for various applications ^22,23^. The rapid development of global industry has led to environmental problems such as weather pollution, emission of toxic gases, and volatile organic compounds, which can pose a threat to human health and the environment^24^. Semiconductor gas sensor technology plays a vital role in detecting these pollutants due to its small size, low cost, and easy manufacturing characteristics. The use of porous silicon in gas sensor technology has improved the accuracy of gas detection by increasing surface reactions on the material. The morphology of the pores can be easily controlled during fabrication, allowing for the design of specific sensor properties^25,26^.

This paper is dedicated to the obtention of electrodeposited porous silicon thin films by varying HF/ethanol ratios. Our main goal is to offer valuable insights into the 3D nanoscale topography, optical characteristics, and gas sensor properties of these films. As far as current knowledge goes, these aspects have not been thoroughly explored in existing literature. The outcomes of our research yield noteworthy results, holding substantial relevance in the realm of affordable device manufacturing nanotechnology for addressing climate pollution.

## Materials and methods

### Thin films deposition

An electrochemical etching setup was utilized to prepare porous silicon samples. P-type silicon wafers with a thickness of 525 µm, resistivity of 1–10 Ω⋅cm and a (100) orientation were cleaned with 5% hydrofluoric acid, acetone, and DI (Deionized) water. A layer of aluminum was deposited on the backside of the wafers using the electron beam gun method. The cleaned glossy side was used as the anode electrode in the etching configuration, with a platinum wire counter electrode. The space between electrodes was filled with an electrolyte solution of HF/ethanol in different concentration ratio according to Table 1. The electrochemical etching process was carried out for 60 min at a current density of $1.99\,{{mA} \mathord{\left/ {\vphantom {{mA} {cm^{2} }}} \right. \kern-0pt} {cm^{2} }}$.

**Table 1:** Electrolyte characteristics used for different samples.

Si#1:8	Si#1:7	Si#1:6	Si#1:5	Si#1:4	Si#0	Sample number
1:8	1:7	1:6	1:5	1:4	‒	Ratio (HF/ethanol)
60	60	60	60	60	‒	Etching time (min)
1.99	1.99	1.99	1.99	1.99	‒	Current density (mA/cm^2^)

### Characterization of the products

#### Morphological and fractal analysis

We used the MIRA 3 field emission scanning electron microscopy (FESEM) at Razi metallurgical research center to examine the shape and structure of the porous silicon layers we obtained.

The surface morphology of the samples was scrutinized through 3D Atomic Force Microscopy (AFM) topographical maps, employing an atomic force microscope (AFM) in contact mode, specifically the Auto Probe CP instrument from Park Scientific. The AFM images were processed and studied using Gwyddion version 2.59 software (available in http://gwyddion.net/). The height-based parameters like root mean square roughness (Sq), maximum peak height (Sp), maximum pit depth (Sv), and maximum height (Sz) were extracted and analyzed according to ISO 25,178-2: 2012—surface texture: areal^27^.

The surface morphology of the samples was further investigated using Minkowski Functionals (MFs). MFs are a set of mathematical descriptors used to analyze and quantify the geometrical properties and morphology of objects or structures, typically in a spatial or image analysis context^15,16^. Herein, we have computed three known MFs, including Volume (V), Surface Area (S), and Euler-Poincaré Characteristic (χ). V measures the volume or spatial extent of a given structure. In image analysis, it quantifies the amount of space enclosed by the object and is obtained according to Eq. (1), where N represents the total number of pixels, N_white_ represents the number of “white” pixels above the threshold. S represents the boundary or surface that encloses the object. It characterizes the interface between the object and its surroundings and is obtained using the Eq. (2), where N_bound_ represents the number of white‒black pixel boundaries. Finally, χ is a topological descriptor that reflects the connectivity and topology of an object. It is related to the number of enclosed cavities or voids within the object and is computed using the Eq. (3), where C_white_ and C_black_ represent the number of continuous sets of white and black pixels^28^, respectively.

1 $$ V = \frac{{N_{white} }}{N} $$

2 $$ S = \frac{{N_{bound} }}{N} $$

3 $$ \chi = \frac{{C_{white} - C_{black} }}{N} $$

The spatial complexity and other specific nanotexture surface aspects of the samples were investigated using fractal parameters. These parameters include fractal dimension (F_D_), fractal sucoolarity (F_S_), and topographical entropy (E). F_D_ is a mathematical measure used to quantify the level of complexity or irregularity in a fractal object or pattern^29^ and can easily computed from Gwyddion software. In the context of surface analysis, it characterizes the roughness or intricacy of a surface at various scales. F_S_ is a parameter that provides insights into the shape and complexity of a fractal. It quantifies the degree to which a fractal object fills space^30^ and is computed using the Eq. (4), where P_0_ represents the count of filled boxes along each line, P_r_ corresponds to the abscissa of the pressure centroid associated with each occupiable box within the image, and n is associated with the total count of boxes along each line. A higher succolarity value suggests that the fractal occupies space more efficiently or densely, while a lower value indicates a sparser or less efficient filling of space.

4 $${F}_{S}= \frac{\sum_{i=1}^{n}{P}_{r}{(k)P}_{0}(k)}{{P}_{0}(n){\sum }_{k=1}^{n}{P}_{r}(k)}$$

Additionally, the topographical entropy, computed using Shannon entropy^31^, measures the degree of disorder or randomness in the distribution of certain characteristics on a surface. In the context of topography or morphology analysis, it can assess the organization of the topographical profile. A lower surface entropy value typically indicates greater variability or disorder in the surface's characteristics, while a higher value suggests more uniformity or regularity. TE value is computed using the Eq. (5), where ${p}_{ij}$ represents the probability of pixels exhibiting discrepancies or not within the height range of the analyzed dataset. The value of this parameter varies of 0 (perfect non-uniform pattern) to 1 (perfect uniform pattern)^32^. F_S_ and TE parameters were computed using R scripts developed in R language using the RStudio software^33^ (available in https://www.rstudio.com/).

5 $$TE= -\sum_{i=1}^{N}\sum_{j=1}^{N}{P}_{ij}log{P}_{ij}$$

#### Optical analysis

The diffuse reflectance spectroscopy (DRS) was used to investigate the optical properties of the samples. The electrolyte concentration which is a basic factor in uniformity of porous Si samples was optimized. The prepared wafer was then applied in an experimental setup for sensor testing, which included a chamber with the porous Si sample, target vapor, connecting pipes with gas cut-off valves, a DC mini air pump, and a Fluke 289 multimeter for electrical data of sensor response which has been reported previously^34^. All estimations were done at room temperature.

By measuring the reflectance spectrum of the samples in the range of 200 to 1200 nm and using the Kubelka–Munk theory and converting the reflectance of the samples to the Kubelka–Munk function (F (R)) by Eq. (6), we have^35^:

6 $$ F(R) = \frac{{(1 - R)^{2} }}{2R} $$

where R is the reflectance of sample and depends on wavelength. Equation (7) shows the relationship between F(R) and the absorption coefficient (α) as^36^:

7 $$ \alpha = \frac{F(R)}{t} $$

where ‘t’ is the thickness of porous silicon.

To assess the optical bandgap energy (E_g_) dependence on the directly allowed transitions, a plot of (αhν)^2^ versus hν was obtained, which further allowed the calculation of E_g_ via the linear hν intercept. Moreover, we used Eq. (8) to calculate the Urbach energy (E_u_) values.

8 $$ \alpha = \alpha_{0} \exp \left( {\frac{h\nu }{{E_{u} }}} \right) $$

where E_u_ is the latitude of the local state. If we plot lnα as photon energy, the amount of that energy can be determined from the slope of the curve.

Finally, the steepness parameter σ, characterizes the broadening of the optical absorption edge due to the electron‒phonon interactions and is calculated by the Eq. (9):

9 $$ \sigma = \frac{{k_{B} T}}{{E_{u} }} $$

where *k*_*B*_ is the Boltzmann constant and *T* is the absolute temperature in K. Therefore, the values of the strength of the electron–phonon interaction (*E*_*e−p*_) can be estimated by the Eq. (10) [36]:

10 $$ E_{e - p} = \frac{2}{3\sigma } $$

### Statistical analysis

The statistical analysis of the data in this study was conducted using the Origin software. To assess the significance of differences among average value of the height-based parameters of porous silicon surfaces, the Analysis of Variance (ANOVA) and Tukey tests were employed. The threshold for statistical significance was set at a *p*-value of 0.05.

## Results and discussion

### Morphological and spatial analysis

The SEM technique offers a formidable magnification capability of up to 1,000,000 times, allowing examination at the nanometer scale^37^. Its significant depth of field is advantageous, enabling simultaneous focus on the specimen’s surface regardless of its roughness^38^. Moreover, SEM surpasses surface topography analysis^39^, offering insights into chemical composition^40^, crystal structure^41^, and electrical properties^42^ of the sample. Enhanced confidence in analysis is achievable by seamlessly switching between various imaging techniques, facilitating cross-correlation of gathered information. Additionally, this method presents a key benefit by generating high-resolution images characterized by substantial pixel density, owing to its impressive resolution.

**Figure 1:**
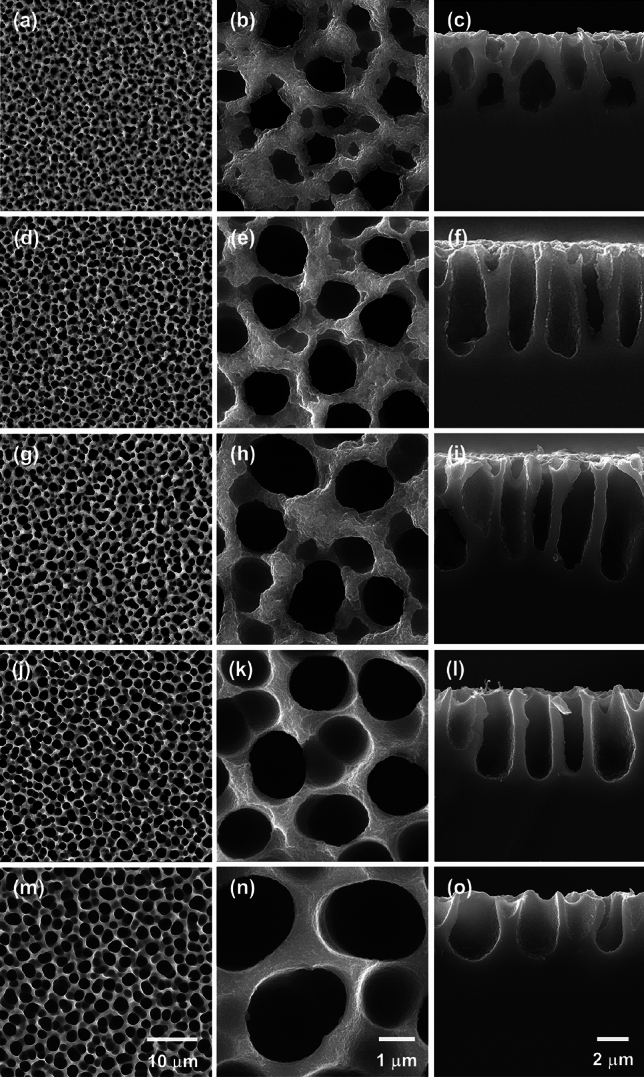
SEM micrographs of the (**a**–**b**) Si#1:4, (**d**–**e**) Si#1:5, (**g**–**h**) Si#1:6, (**j**–**k**) Si#1:7, and (**m**–**n**) Si#1:8 porous silicon surfaces using magnifications of 15,000 × and 35,000x. On the right, correspondent cross section images (15,000x).

As mentioned in the methodology of this work, the conditions of the etching process were maintained, only varying the HF/ethanol ratio to produce the samples. In our study, to demonstrate how alterations in this ratio impact the micromorphology of the samples, SEM micrographs displaying surface topographies of the films are depicted in Fig. 1. Within this figure, at magnifications of 15,000 × and 35,000x, discernible modifications on the surface are evident, showcasing considerable variations in pore sizes distributed across all surfaces within each sample.

Representative images of the surface of the samples and also cross section analysis results illustrate that wear produces porous structures and a significant increase in the average diameter of the pores can be observed, which varied between 1.02 ± 0.09 μm and 2.35 ± 0.11 μm, for Si#1:4 and Si#1:8, respectively. Samples Si#1:5, Si#1:6 and Si#1:7 reached values of the order of 1.19 ± 0.14, 1.39 ± 0.13 and, 1.52 ± 0.06 μm, respectively. On the other hand, the cross-sectional images showed a compensation, in which it is possible to observe a decrease in pore depth, varying between 4.11 ± 0.24 and 6.87 ± 0.57 for Si#1:8 and Si#1:5, with the smallest and largest values, respectively. For the other films, the average depths were Si#1:4 = 4.26 ± 0.11, Si#1:6 = 5.63 ± 0.44, and Si#1:7 = 5.84 ± 0.24. These results demonstrate that in the acid etching process, both the diameter and depth of the pores vary depending on the HF/ethanol concentration.

**Figure 2:**
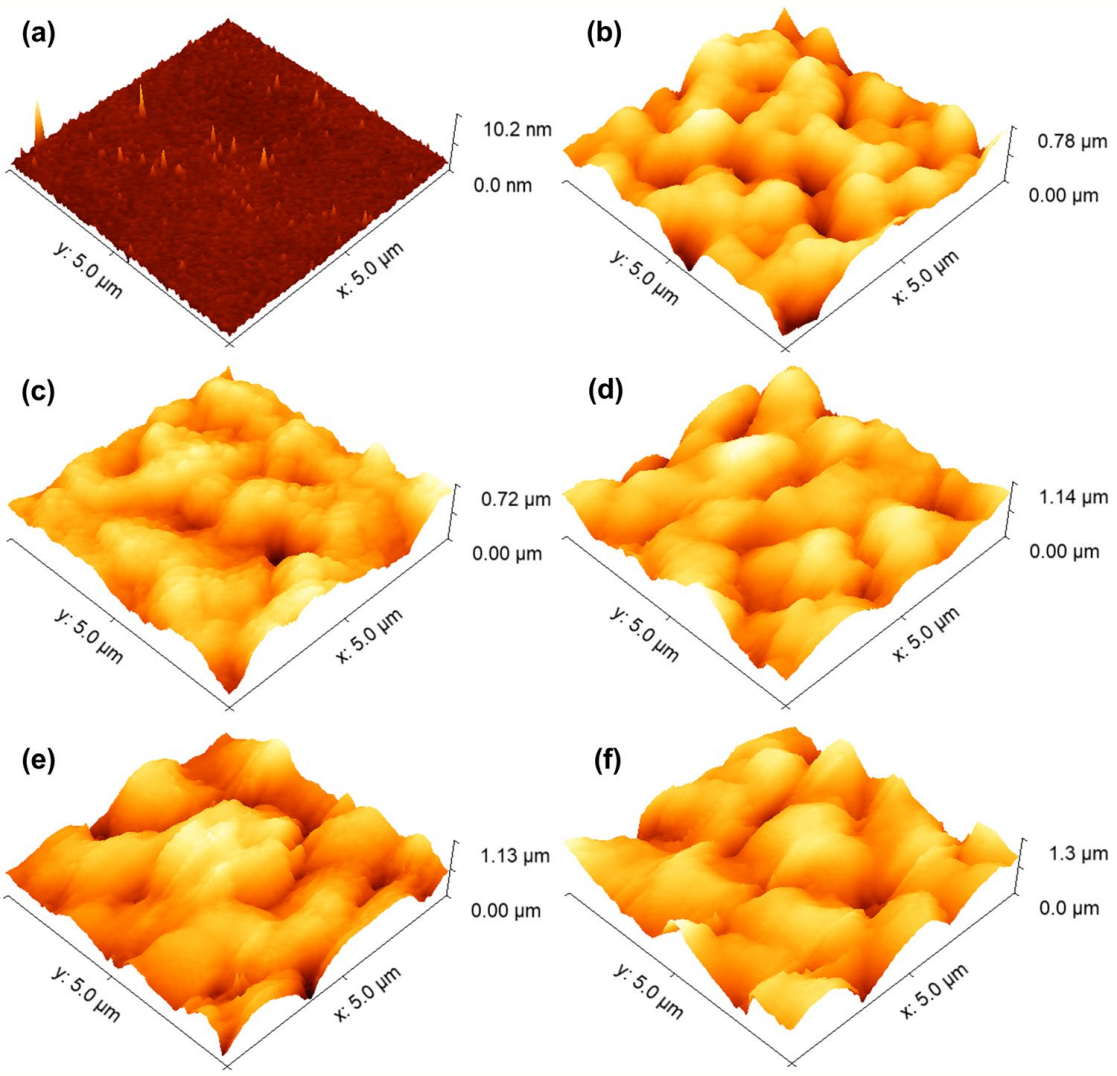
Representative 3-D AFM micrographs of (**a**) Si#0, (**b**) Si#1:4, (**c**) Si#1:5, (**d**) Si#1:6, (**e**) Si#1:7, and (**f**) Si#1:8 porous silicon surfaces.

The morphological characteristics of the samples were also analyzed via AFM, and their 3D surface representations are depicted in Fig. 2. Figure 2a illustrates the surface morphology of the pure Si sample (Si#0), revealing a smooth surface with minor irregularities observable over a 10.2 nm Z‒scale. Conversely, the samples prepared using varying HF/ethanol ratios (Fig. 2a‒f) exhibit a similar surface morphology, albeit notably rougher when compared to the Si#0 sample. These surfaces are predominantly characterized by rugged, mountainous regions displaying substantial irregularities. Such pronounced surface irregularity signifies that the surface porosity of these specimens consistently exceeds that of the pristine Si#0 sample.

Table 2 presents the topographical parameters associated with each sample. As evident, the Si#0 sample boasts a root mean square surface roughness (Sq) of a mere 0.3 nm, which corroborates our qualitative assessment based on Fig. 2a. In stark contrast, the samples produced under a HF/ethanol solution exhibit average roughness values spanning the range of 113 nm (Si#1:4) to 218 nm (Si#1:8), markedly higher than the Si#0 sample. The other height-based parameters, such as maximum peak height (Sp), maximum pit depth (Sv), and maximum height (Sz), follow a similar trend. This underscores the absolute influence of the HF/ethanol ratio on the vertical growth of the samples across various scales. In this regard, it is noteworthy that the roughness values ascend from sample Si#1:4 to Si#1:8, underscoring the pivotal role played by the HF/ethanol ratio in shaping the 3D spatial patterns on the porous silicon surfaces.

**Table 2:** Height parameters of electrodeposited porous Si surfaces.

Parameter	Unit	Si#0	Si#1:4	Si#1:5	Si#1:6	Si#1:7	Si#1:8
Sq	[nm]	0.3 ± 0.1	113.3 ± 18.0	118.2 ± 24.8	188.5 ± 37.9	196.0 ± 33.1	218.4 ± 33.3
Sp	[nm]	11.4 ± 5.9	309.3 ± 26.3	333.0 ± 48.0	518.0 ± 74.5	581.0 ± 95.7	563.0 ± 43.3
Sv	[nm]	1.5 ± 0.4	351.0 ± 108.7	526 .2 ± 163.2	922.3 ± 309.6	697.7 ± 114.9	890.0 ± 102.1
Sz	[nm]	12.9 ± 6.3	771.3 ± 153.1	859.2 ± 196.0	1440.0 ± 380.5	1279.0 ± 131.0	1453.0 ± 127.5

**Figure 3:**
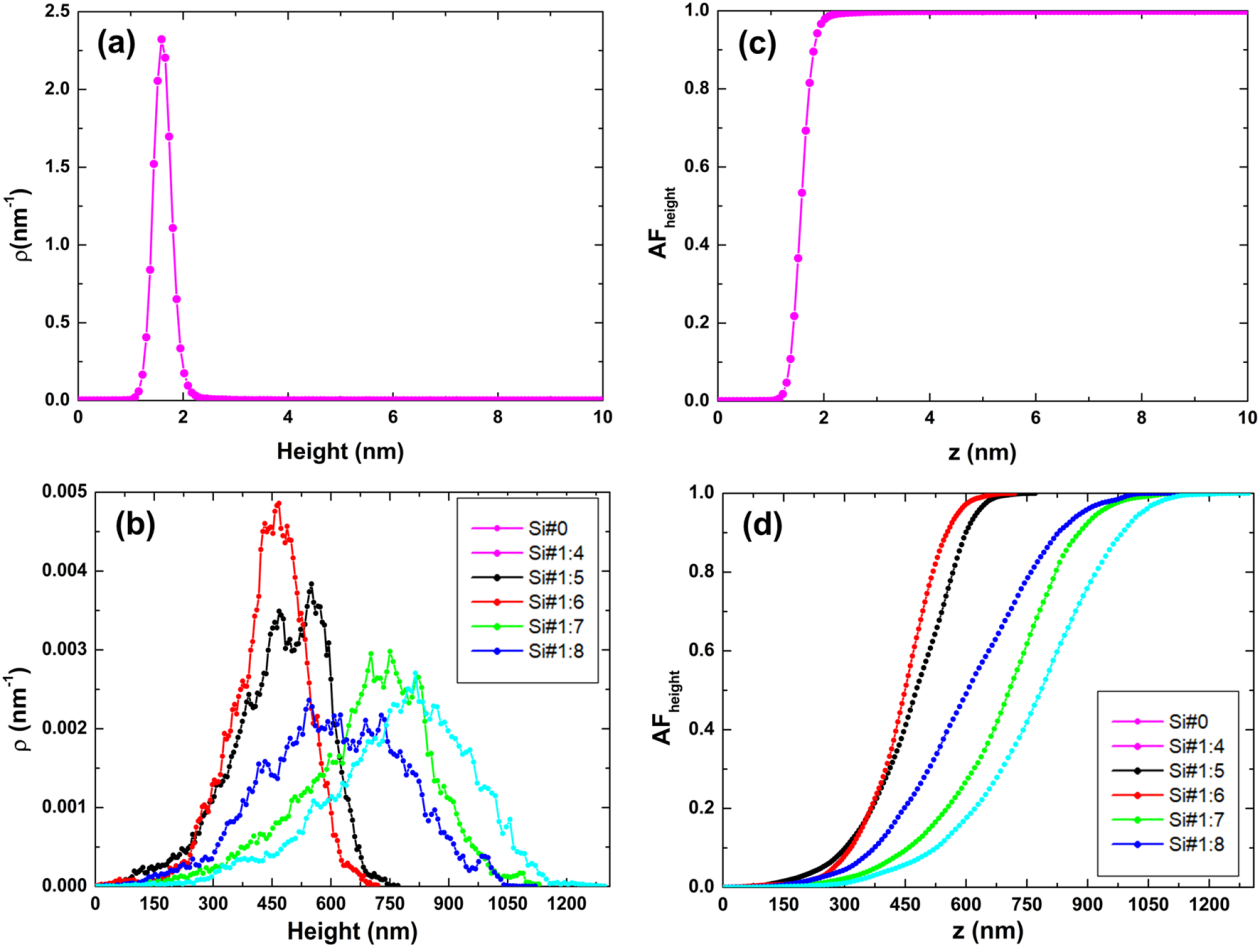
(**a**)‒(**b**) Topographical profile and (**c**)‒(**d**) Abbott-Firestone curves of electrodeposited porous Si surfaces.

Figure 3 illustrates the topographic profiles of the samples. As evident, the Si#0 sample's distribution of rough peaks is notably concentrated within the 1‒2 nm range (Fig. 3a). Moreover, this sample displays an exceptionally narrow distribution, indicative of a leptokurtic surface profile^43^. On the other hand, the samples subjected to HF/ethanol treatment present broader distributions of rough peaks, spanning a range from 150 to 1200 nm. This unequivocally confirms that HF/ethanol different ratios have altered the surface characteristics, fostering vertical profile growth through acid‒induced modification. Notably, starting from a HF/ethanol ratio of 1:6 (Si#1:6), the distributions exhibit increased flatness, signifying more platykurtic surface profiles^31^. Furthermore, when examining the Abbott-Firestone curves (AFC)^44,45^, it becomes evident that the Si#0 sample's curve (Fig. 3c) rapidly approaches its maximum compared to the samples obtained with the HF/ethanol solution (Fig. 3d). This observation underscores that the height distribution of the Si#0 sample does not align with the centering observed in samples Si#1:4 to Si#1:8. As a consequence of the analogous distribution shape seen in samples Si#1:4 to Si#1:8, it's worth noting that their AFCs exhibit the typical S-shaped profile^10^.

**Figure 4:**
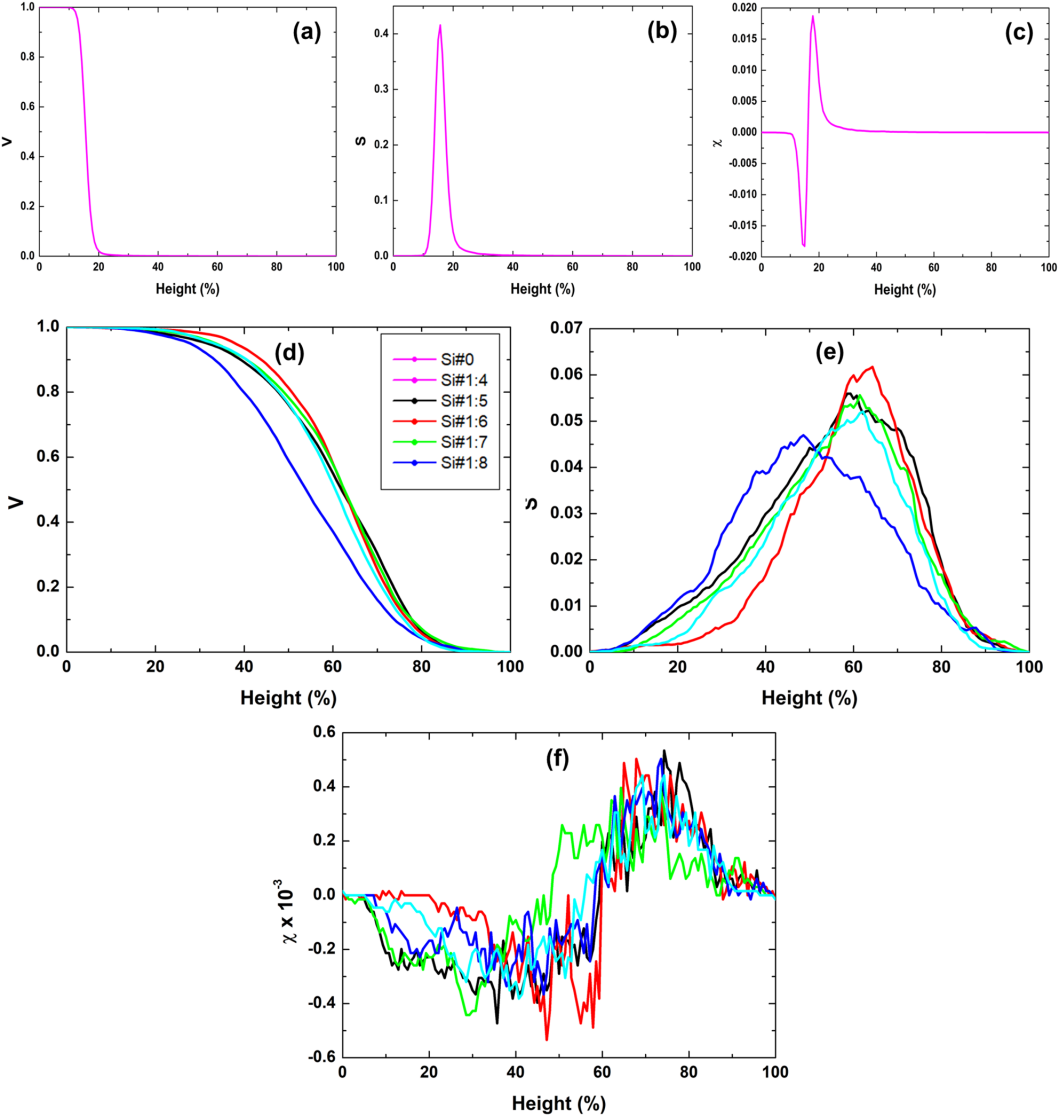
Minkowski volume, Minkowski boundary, and Minkowski connectivity of electrodeposited porous Si surfaces.

We delved further into the morphological characteristics of the samples using Minkowski Functionals (MFs)^12,13^, and the results are depicted in Fig. 4. In Fig. 4a, we can observe that the Minkowski volume (V) of the Si#0 sample diminishes as relative height (%) increases. However, V consistently diminishes with relative height (%) across all silicon surfaces Si#1:4 to Si#1:8 (Fig. 3d), indicating a substantial portion of material existing above a specific relative height (%) for these samples compared to the Si#0 sample. This robustly supports the notion of increased surface porosity along the surfaces of the samples treated with the HF/ethanol solution. Figure 4) illustrates the Minkowski boundary (S) curve for the Si#0 sample, displaying a narrow non-monotonic trend^10^ with relative height, centered below 20%. In contrast, samples Si#1:4 to Si#1:8 exhibit maximum S values with relative height centered above 20%. Furthermore, the peak values of S do not demonstrate a linear increase relative to the HF/ethanol ratio. Notably, samples Si#1:4 and Si#1:7 display the highest and lowest maximum S values, respectively. This underscores that the relative distribution of voids exhibits non‒linear behavior in response to variations in the HF/ethanol ratio. Moving to Fig. 4c, it presents the Euler-Poincaré characteristic (χ) of the Si#0 sample, which adheres to the conventional pattern with distinct negative minima and positive maxima values^46^. These values, when compared to samples obtained with different HF/ethanol ratios, are notably shifted to the left along the relative heights. Moreover, the maximum and minimum χ values for samples Si#1:4 to Si#1:8 are slightly higher than those of the Si#0 sample. It worth noting that no linear behavior was observed in these maximum and minimum values concerning the different HF/ethanol ratios. This suggests that spatial connectivity exhibits non-linear characteristics in response to changes in HF/ethanol solution ratios. Therefore, the results encompassing V, S, and χ collectively emphasize the unique spatial distribution of matter in samples Si#1:4 to Si#1:8 in contrast to the Si#0 sample according to Fig. 4d, e and f respectively. This substantiates that acid etching facilitated the formation of similar highly rough morphologies.

### Fractal analysis of the 3D spatial patterns

It is known that the microtexture of nanoscale surfaces can be completely mapped by fractal parameters^30,47^. Herein, we use the fractal dimension (F_D_), the fractal succolarity (F_S_), and the topographic entropy (TE) to evaluate the complexity, surface porosity, and uniformity of the topographic profile of the samples, respectively, as shown in Table 3. The highest F_D_ value was observed for Si#0 sample. There is a discernible decline in the average F_D_ values for samples Si#1:4 to Si#1:8, signifying a reduction in the spatial complexity of the samples as function as HF/ethanol solution ratio employed. Consequently, we can infer that the acid etching process led to the creation of less intricate surfaces with shorter-range correlations, despite their increased roughness. This indicates that as the HF/ethanol solution ratio was changed from 1/4 to 1/8, the surfaces progressed towards an almost perfectly flat state (FD = 2)^18^.

**Table 3:** Fractal parameters of porous silicon surfaces.

Parameter	Samples					
	Si#0	Si#1:4	Si#1:5	Si#1:6	Si#1:7	Si#1:8
FD	2.276 ± 0.053	2.2 ± 0.016	2.182 ± 0.017	2.178 ± 0.019	2.180 ± 0.011	2.173 ± 0.007
FS	0.438 ± 0.017	0.452 ± 0.004	0.464 ± 0.033	0.470 ± 0.022	0.482 ± 0.016	0.535 ± 0.002
TE*	0.969 ± 0.002	0.985 ± 0.007	0.984 ± 0.011	0.959 ± 0.016	0.986 ± 0.009	0.984 ± 0.007

*Samples without significant difference ANOVA One-Way and Tukey Test (*p* < 0.05).

The assessment of surface porosity using the fractal sucoolarity^30^ parameter reveals that the Si#0 surface is the less porous surface, characterized by the lowest FS value (~ 0.44). Furthermore, porosity consistently increases (with statistical significance at *p* < 0.05) from samples Si#1:4 to Si#1:8, ranging from ~ 0.45 to ~ 0.54. This signifies that surface porosity escalates as the HF/ethanol solution ratio decreases. Hence, the sample produced under a 1/8 ratio proved to be the most porous among all, in alignment with our qualitative analysis of the AFM images. It’s noteworthy that the elevation in surface roughness resulted in flatter surfaces with a more extensive distribution of regions permitting fluid access from lower to upper bands. This finding aligns with the behavior of the Urbach energy, which exhibits an increase from samples Si#1:4 to Si#1:8, reflecting the rise in surface porosity across the samples. Additionally, we assessed the uniformity of the topographic profiles using topographic entropy (TE)^32^. Notably, the Si#0 surface, along with the sample produced with an HF/ethanol ratio of 1/6, displayed the lowest average TE values. However, our statistical analysis revealed that the average values, in general, do not exhibit statistically significant differences (*p* > 0.05). In simpler terms, the tails of the distributions (for N = 3) overlap, demonstrating that any mean value is a valid representation. Consequently, we can conclude that all topographical patterns, including those of the Si#0 sample, demonstrate similar levels of topographical uniformity. This implies that they possess comparable proportions of uniform and non-uniform patterns. It's worth emphasizing that the E values approximating 1 for all samples indicate a high degree of uniformity in their topographical patterns, affirming the excellent quality and robustness of our samples.

### Optical properties

**Figure 5:**
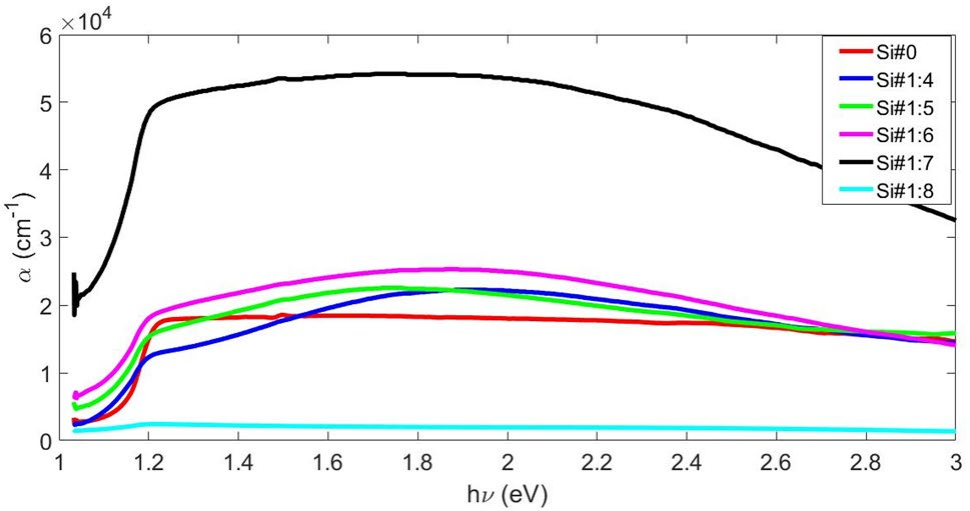
Adsorption coefficient of Si surfaces with different porosity.

**Figure 6:**
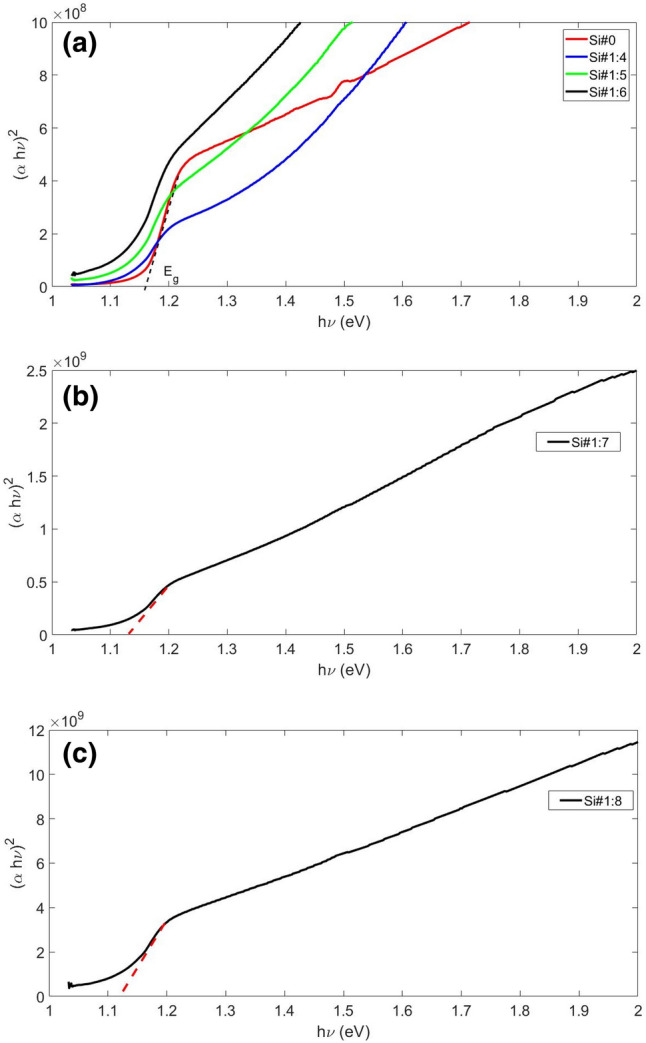
Diagram of (αhν)^2^ versus hν and determination E_g_ from (**a**) Si#1:4 to Si#1:6, (**b**) Si#1:7, and (**c**) Si#1:8.

In Fig. 5, the absorption diagram is drawn in terms of incident photon energy. The absorption of the samples has increased with the increase of porosity up to sample Si#1:7 and the porosity is 54%, but in the Si#1:8 sample it has decreased drastically, and it is even less than non‒porous silicon surface (Si#0). Accordingly, we chose to check the behavior of the sensor. The E_g_ values (Fig. 6) decrease smoothly from 1.18 eV to 1.12 eV as the amount of porosity increases, as shown in Table 4. By increasing the amount of porosity, the E_g_ has decreased, that is, the number of localized states and defects has increased, which is exactly confirmed by calculating the Urbach energy (E_u_), because with the increase in porosity, the amount of E_u_ has increased (Table 4).

**Table 4:** The values of optical parameters of porous Si surfaces.

Optical parameter	Si#0	Si#1:4	Si#1:5	Si#1:6	Si#1:7	Si#1:8
E_g_ (eV) (optical bandgap energy)	1.18	1.16	1.15	1.14	1.13	1.12
E_u_ (meV) (Urbach energy)	69	72	90	152	156	314
σ (steepness parameter)	0.37	0.35	0.28	0.17	0.16	0.08
Ee–p (electron–phonon interaction)	1.78	1.86	2.33	3.94	4.04	8.13

**Figure 7:**
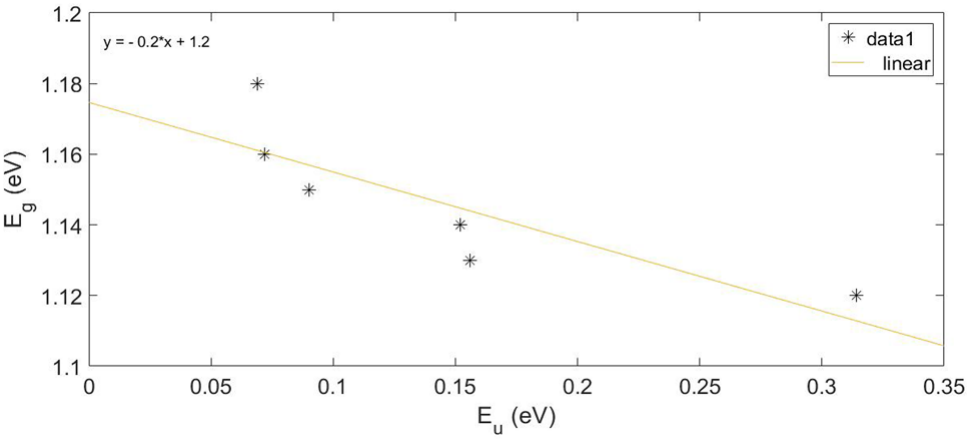
The relation between Eg and width of E_u_ of Si porous surfaces.

As can be seen, the porosity of the surfaces has created holes and defects in the structure also increased the density of replaced levels, as verified by the E_g_ and E_u_ values shown in Table 4. Notably, we can note that E_u_ value increases with increasing porosity. Furthermore, there is a good linear relation between E_g_ and E_u_, as shown in Fig. 7. The empirical formula for this linear fit is given by Eq. (11). The average value of the constant in Eq. (11) is also obtained from the linear fit calculated down to 1.2 eV ignoring tailing. Curiously, a linear relationship between the E_g_ and the width of the E_u_ has also been observed in other semiconductors^48^.

11 $$ E_{g} = 1.2 - 0.2E_{u} $$

The estimated values of steepness parameters and the strength of the electron‒phonon interaction are listed in Table 4. The porosity of the Si structure affects the E_u_ emission, and the higher the porosity, the stronger the E_u_ emission. Porosity in the Si structure increases the number of localized states, vacancies, and dislocation defects. We estimated the values ​​of the steepness parameter and the strength of the electron‒phonon interaction according to Table 2. The E_u_ and Ee-*p* values ​​increase with increasing Si porosity. On the other hand, the steepness parameter decreases. Hence, increased porosity diminishes steepness values due to heightened dislocations and structural disturbances. Porosity reduces the transconductance parameter while augmenting electron‒phonon interaction strength.

### Evaluating the sensing properties

**Figure 8:**
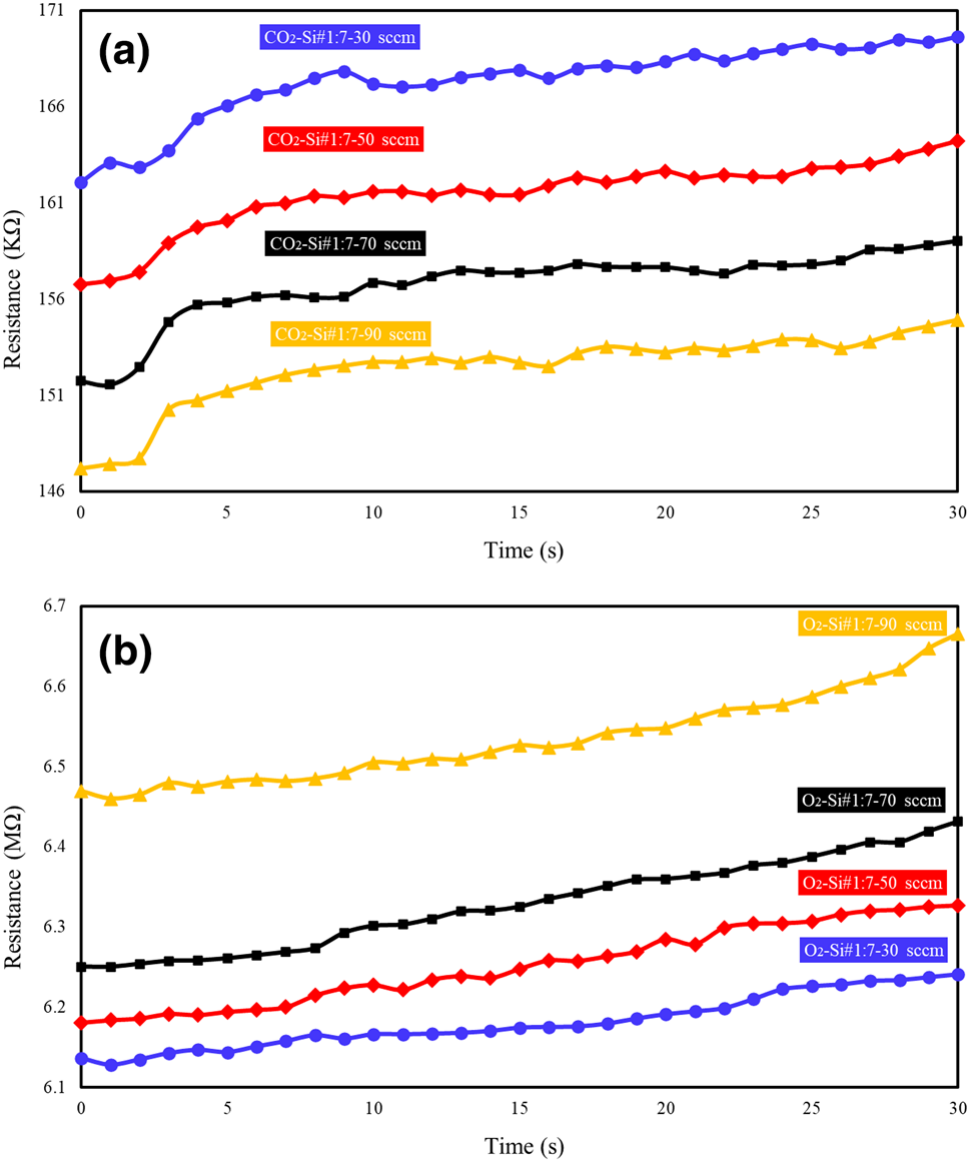
Experimental value of the resistance-based gas sensors to (**a**) CO_2_ (**b**) O_2_ at 20 °C and different gas concentrations (30, 50, 70 and 90 sccm).

Figure 8 shows the experimental values of the resistance-based gas sensors regarding to CO_2_ and O_2_ at 20 °C with different gas concentrations (30, 50, 70 and 90 sccm). Figure 8a shows the changes in electrical resistance of the gas sensor in terms of time and in different concentrations (30, 50, 70, and 90 sccm) of CO_2_ gas. As can be seen, the electrical resistance increases with the absorption of CO_2_ gas by the sensor. The electrical resistance with a fixed concentration of 30 sccm of CO_2_ gas increases from 162 to 169.6 KΩ for 30 s, and for a concentration of 90 sccm, the electrical resistance increases from 147 to 154.9 KΩ. The results of Fig. 8a show that the electrical resistance has decreased with the increase in the concentration of CO_2_ gas. With the increase of CO_2_ gas concentration from 30 to 90 sccm, the electrical resistance has decreased from 162 to 147 KΩ respectively. Comparison between the fabricated gas sensor and other works shows good agreement. Abdali et al^49^. show that for the CO_2_ gas sensor with different concentrations, the electrical resistance has decreased with increasing gas concentration. In Fig. 8b, the diagram of changes in the electric resistance of the sensor in terms of time and in different concentrations (30, 50, 70 and 90 sccm) of O_2_ gas has been examined. As can be seen from Fig. 8b, the electrical resistance increases with the absorption of O_2_ gas by the sensor and the electrical resistance with fixed concentrations of 30 sccm and 90 sccm increases from 6.14 to 6.24 MΩ and from 6.47 to 6.67MΩ, respectively, with the passage of time of 30 s. The results from Fig. 8b show that unlike the behavior of sensor in the presence of CO_2_ gas, with the increase in concentration of O_2_ gas from 30 and 90 sccm, the electrical resistance has increased from 6.14 to 6.47 MΩ. A comparison between the graphs in Fig. 8a and b shows that the electrical resistance of gas sensor in the presence of CO_2_ and O_2_ gases at a concentration of 30 sccm are 162 KΩ and 6.14 MΩ, respectively, which indicate the optimal response of the gas sensor.

Resistance-based gas sensor response is obtained from the following Equation^50^:

12 $$ {\text{Re}} sponse(\% ) = \frac{{R_{g} - R_{0} }}{{R_{g} }} \times 100 = \frac{\Delta R}{R}(\% ) $$

**Figure 9:**
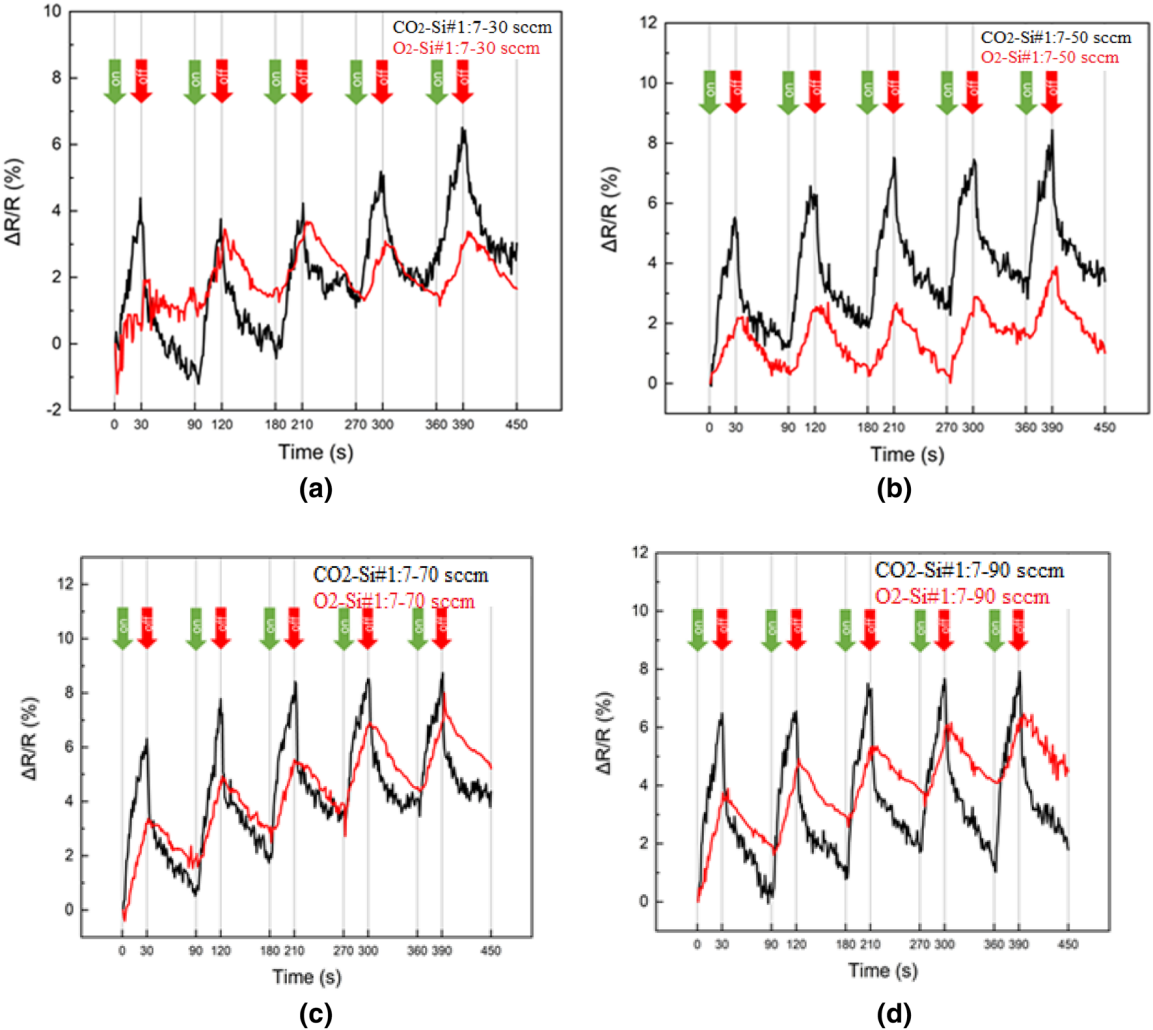
Response (%) of CO_2_ and O_2_ gas sensors in different gas concentration (**a**) 30 sccm (**b**) 40 sccm (**c**) 70 sccm (**d**) 90 sccm.

In this regard, R_g_ is the electrical resistance of the sensor in the presence of CO_2_ and O_2_ gas. As can be seen from Fig. 8, the response of the resistance-based sensor in different concentrations is higher for CO_2_ gas. Also, the results from Fig. 9 show that with the increase in the concentration of CO_2_ and O_2_ gases from 30 to 90 sccm, the response of the sensors has increased from 4 to 8% and from 1 to 6%, respectively. The response time of the sensor (t 90%) is defined as the time it takes to reach 90% of the final value, and the reversibility time (t 10%) is the time from 90 to 10% of the initial value^51^. As can be seen from Fig. 9, the response time of the CO_2_ gas sensor is equal to 27 s and the reversibility time is 81 s. The response and reversibility times for the O_2_ gas sensor are 29 s and 83 s, respectively.

The comparison between CO_2_ and O_2_ gas sensor performance is shown in Table 5. In Ref.^52^, a resistance-based sensor for detecting CO_2_, H_2_, and C_2_H_2_ gases using nanoporous graphene has been investigated. That article showed that at concentration of 60 sccm, the response of the sensor to CO_2_, H_2_ and C_2_H_2_ gases is 37.04%, 16.16% and 2.87%, respectively. The CO_2_ gas resistance-based sensor based on MgFe_2_O_4_ shows response time of 3 s at temperature of 300 °C^53^. The comparison between CO_2_ and O_2_ resistance-based gas sensors based on CNTs, ZnO and SnO_2_ shows the response time and reversibility in a few minutes^54–56^. Smith et. al.^57^ investigated a resistance-based CO_2_ gas sensor based on graphene. They showed that at room temperature and at a concentration of 400 ppm, the response time of the sensor is 3 s. The comparison between the response time and reversibility of the fabricated sensor with other CO_2_ and O_2_ gas resistance-based sensors shows that the fabricated sensor in the present work has a high response speed at room temperature ^57–59^.

**Table 5:** Comparison of CO_2_ and O_2_ gas sensors.

Gas/material	Temperature	Gas concentration	Mechanism	Response time	Recovery time	Response	Refs
	(°C)	–	–	(s)	(s)	%	–
CO_2_/Porous Si	RT	30 sccm	Resistance	27	81	6	This work
O_2_/Porous Si	RT	30 sccm	Resistance	29	83	3	This work
CO_2_/nanoporous graphene	RT	30 sccm	Resistance	60	< 700	10*	^52^
CO_2_/La_2_O_3_–SnO_2_	350	1000 ppm	Resistance	> 15 min	> 15 min	2.5*	^53^
CO_2_/MgFe_2_O_4_	300	5000 ppm	Resistance	120	240	36	^54^
CO_2_/CNTs	RT	1–50%	Resistance	> 400	> 400	10*	^55^
O_2_/ZnO	350	100 ppm	Resistance	240	300	–	^56^
CO_2_/Graphene	RT	400 ppm	Resistance	3	–	0.00364*	^57^
CO_2_/PEI**–**Cr_2_O_3_	RT	1000 ppm	Resistance	20	22	–	^58^
CO_2_/Bi_2_O_3_	RT	100 ppm	Resistance	132	82	179	^59^

## Conclusions

In summary, P-type silicon with a (100) orientation served as the substrate for porous silicon fabrication through the electrochemical etching method, utilizing HF/ethanol electrolyte at varying ratios. Characterizations via AFM, SEM, and DRS yielded essential insights into 3D topography, morphological, and optical properties of the thins films. Morphological analysis underscored the formation of unique surfaces with distinct morphologies compared to untreated samples, fostering substantially rougher surfaces with maintained flat characteristics, as confirmed by Minkowski Functionals analysis. Fractal mathematics exploration of 3D spatial patterns emphasized that HF/ethanol-treated surfaces, despite increased roughness, exhibited flatter attributes compared to untreated Si samples. Fractal analysis highlighted roughness as a fundamental factor in creating more porous surfaces, evident in the fractal juice parameter. Topographic entropy assessments demonstrated similar high-quality attributes in the uniformity of 3D topographic patterns across all samples. Optical calculations revealed that increased porosity correlated with a rise in localized states and defects, alongside an augmentation in the E_u_ value. Consequently, porosity contributed to a reduction in steepness values due to heightened dislocations and structural disturbances. The transconductance parameter decreased, while the strength of electron–phonon interaction increased with porosity. Subsequently, the porous sample was employed as a gas sensor for CO_2_ and O_2_ vapors at room temperature, monitoring changes in electrical resistance over time and varying concentrations. Increasing CO_2_ gas concentration from 30 to 90 sccm resulted in a decrease in electrical resistance from 162 KΩ to 147 KΩ, with a response time of 27 s and a reversibility time of 81 s. For the O_2_ gas sensor, response and reversibility times were 29 s and 83 s, respectively. These findings underscore the pivotal role of surface porosity in shaping the optical and sensing properties of silicon porous surfaces under different HF/ethanol ratios.

## Data Availability

The data that support the findings of this study are available from the corresponding author upon reasonable request.

## References

[1] Uhlir A (1956). Electrolytic shaping of germanium and silicon. Bell. Syst. Tech. J..

[2] Lachenani H, Larabi A, Gabouze N (2019). Study of structural, electronic and vibrational properties of porous silicon with different porosity. Silicon.

[3] Solaymani S, Ghaderi A, Dejam L, Garczyk Z, Sapota W, Stach S, Dalouji V, Luna C, Elahi SM, Elahi SH (2017). Correlation between the multifractal structure, crystalline and photoluminescence properties of engineered CZO thin films. Int. J. Hydrog. Energy.

[4] Praveenkumar S, Lingaraja D, Mahiz Mathi P, Dinesh Ram G (2019). An experimental study of optoelectronic properties of porous silicon for solar cell application. Optik (Stuttg)..

[5] Dalouji V, Elahi SM, Ghaderi A, Solaymani S (2016). Influence of annealing temperature on berthelot-type hopping conduction mechanism in carbon-nickel composite films. Chin. Phys. Lett..

[6] Russo L, Colangelo F, Cioffi R, Rea I, De Stefano L (2011). A mechanochemical approach to porous silicon nanoparticles fabrication. Materials (Basel).

[7] Bahar M, Gholami M, Azim-Araghi ME (2014). Sol–gel synthesized Titania nanoparticles deposited on porous polycrystalline silicon: Improved carbon dioxide sensor properties. Mater. Sci. Semicond. Process..

[8] Myers P, Michael J (2013). Sailor: Porous silicon in practice. Preparation, characterization and applications. Chromatographia.

[9] Levitsky I (2015). Porous silicon structures as optical gas sensors. Sensors.

[10] Matos RS (2023). Evaluating the roughness dynamics of kefir biofilms grown on Amazon cupuaçu juice: A monofractal and multifractal approach. Microscopy.

[11] Ţălu Ş, Solaymani S, Bramowicz M, Naseri N, Kulesza S, Ghaderi A (2016). Surface micromorphology and fractal geometry of Co/CP/X (X = Cu, Ti, SM and Ni) nanoflake electrocatalysts. RSC Adv..

[12] Lighvan YL (2022). Morphological characteristics and Minkowski functionals of Ag-DLC thin films. Vak. Forsch. Prax..

[13] Grayeli Korpi A (2019). Minkowski functional characterization and fractal analysis of surfaces of titanium nitride films. Mater. Res. Express.

[14] da Fonseca de Albuquerque MD (2023). Effect of self-bias voltage on the nanoscale morphological properties of corn starch-based films modified by hexamethyldisiloxane plasma. Plasma Process. Polym..

[15] Pinto EP (2023). Nanoscale 3D spatial analysis of zirconia disc surfaces subjected to different laser treatments. Fractal Fract..

[16] Matos RS (2022). Percolative, multifractal, and symmetry properties of the surface at nanoscale of Cu-Ni bimetallic thin films deposited by RF-PECVD. Symmetry (Basel)..

[17] Shakoury R (2023). Investigation of deposition temperature effect on spatial patterns of MgF_2_ thin films. Microsc. Res. Tech..

[18] Zelati A (2023). Morphological and multifractal properties of Cr thin films deposited onto different substrates. Microsc. Res. Tech..

[19] Casalino M, Coppola G, Iodice M, Rendina I, Sirleto L (2010). Near-infrared sub-bandgap all-silicon photodetectors: State of the art and perspectives. Sensors.

[20] Canham LT (1990). Silicon quantum wire array fabrication by electrochemical and chemical dissolution of wafers. Appl. Phys. Lett..

[21] Stutzmann M, Weber J, Brandt MS, Fuchs HD, Rosenbauer M, Deak P, Höpner A, Breitschwerdt A (1992). Visible luminescence from silicon. Adv. Solid State Phys..

[22] Salih E, Ayesh AI (2020). CO, CO_2_, and SO_2_ detection based on functionalized graphene nanoribbons: First principles study. Phys. E Low-Dimens. Syst. Nanostruct..

[23] Ghaderi A (2022). Advanced microstructure, morphology and CO gas sensor properties of Cu/Ni bilayers at nanoscale. Sci. Rep..

[24] Gosteva EA, Rubtsova KI, Silina MD, Starkov VV (2020). Formation of gradient-porous silicon structures with variable pore morphology. Mech. Solids.

[25] Wang J, Zhou Q, Peng S, Xu L, Zeng W (2020). Volatile organic compounds gas sensors based on molybdenum oxides: A mini review. Front. Chem..

[26] Thomas T (2021). Porous silicon/α-MoO_3_ nanohybrid based fast and highly sensitive CO_2_ gas sensors. Vacuum.

[27] Leach R (2013). Characterisation of Areal Surface Texture Characterisation of Areal Surface Texture.

[28] Nečas D, Klapetek P (2012). Gwyddion: An open-source software for SPM data analysis. Cent. Eur. J. Phys..

[29] Ţălu Ș (2015). Micro and nanoscale characterization of three dimensional surfaces.

[30] Ţălu Ş, Abdolghaderi S, Pinto EP, Matos RS, Salerno M (2020). Advanced fractal analysis of nanoscale topography of Ag/DLC composite synthesized by RF-PECVD. Surf. Eng..

[31] Dejam L (2023). Advanced nano-texture, optical bandgap, and Urbach energy analysis of NiO/Si heterojunctions. Sci. Rep..

[32] Matos RS (2018). Superficial characterization of kefir biofilms associated with Açaí and Cupuaçu extracts. Arab. J. Sci. Eng..

[33] Racine JS (2012). RStudio: A platform-independent IDE for R and Sweave. J. Appl. Econom..

[34] Moghimi E, Azim Araghi ME (2023). Ethanol and acetone gas sensor properties of porous silicon based on resistance response. Silicon.

[35] Hosseini S, Dejam L, Elahi H (2022). The characterization of amorphous AZO-n/Si-p hetrojunction diode for solar cell application. Opt. Quantum Electron..

[36] Ghaderi A (2023). Evaluating structural, morphological, and multifractal aspects of n-ZnO/p-ZnO homojunctions and n-ZnO/p-NiO heterojunctions. Microsc. Res. Tech..

[37] Hoseinzadeh T, Solaymani S, Kulesza S, Achour A, Ghorannevis Z, Ţălu Ș, Bramowicz M, Ghoranneviss M, Rezaee S, Boochani A, Mozaffari N (2018). Microstructure, fractal geometry and dye-sensitized solar cells performance of CdS/TiO2 nanostructures. J. Electroanal. Chem..

[38] Méndez-Albores A, González-Arellano SG, Reyes-Vidal Y, Torres J, Ţălu Ş, Cercado B, Trejo G (2017). Electrodeposited chrome/silver nanoparticle (Cr/AgNPs) composite coatings: Characterization and antibacterial activity. J. Alloys Compd..

[39] Tofel P, Částková K, Říha D, Sobola D, Papež N, Kaštyl J, Ţălu Ş, Hadaš Z (2022). Triboelectric response of electrospun stratified PVDF and PA structures. Nanomaterials.

[40] Ghobadi N, Hafezi F, Naderi S (2019). Microstructure and optical bandgap of cobalt selenide nanofilms. Semiconductors.

[41] Naseri N, Ţălu Ş, Kulesza S, Qarechalloo S, Achour A, Bramowicz M, Ghaderi A, Solaymani S (2018). How morphological surface parameters are correlated with electrocatalytic performance of cobalt-based nanostructures. J. Ind. Eng. Chem..

[42] Černohorský P, Pisarenko T, Papež N, Sobola D, Ţălu Ş, Částková K, Kaštyl J, Macků R, Škarvada P, Sedlák P (2021). Structure tuning and electrical properties of mixed PVDF and nylon nanofibers. Materials.

[43] Matos RS, Ramos GQ, da Fonseca Filho HD, Ţălu Ş (2020). Advanced micromorphology study of microbial films grown on Kefir loaded with Açaí extract. Micron.

[44] Abbott EJ, Firestone FA (1933). Specifying surface quality. Mech. Eng..

[45] Schmähling J, Hamprecht FA (2007). Generalizing the Abbott-Firestone curve by two new surface descriptors. Wear.

[46] Ramos GQ (2023). SEM-imaging-based mapping of monofractal and multifractal patterns of the Piper krukoffii Yunck leaf surface architecture. Flora.

[47] Matos RS (2021). Correlating structure and morphology of andiroba leaf (*Carapa guianensis* Aubl.) by microscopy and fractal theory analyses. Appl. Sci..

[48] Dejam L (2023). ZnO, Cu-doped ZnO, Al-doped ZnO and Cu-Al doped ZnO thin films: Advanced micro-morphology, crystalline structures and optical properties. Results Phys..

[49] Abdali H, Heli B, Ajji A (2019). Cellulose nanopaper cross-linked amino graphene/polyaniline sensors to detect CO_2_ gas at room temperature. Sensors.

[50] Bagheri F, Haratizadeh H (2022). UV-activated CO_2_ sensor based on ZnO nanoparticles at low temperatures. Mater. Sci. Semicond. Process..

[51] Pour BG, Aval FL (2018). Monitoring of hydrogen concentration using capacitive nanosensor in a 1% H2–N2 mixture. Micro Nano Lett..

[52] Shaban M, Ali S, Rabia M (2019). Design and application of nanoporous graphene oxide film for CO_2_, H_2_, and C_2_H_2_ gases sensing. J. Mater. Res. Technol..

[53] Iwata T, Matsuda K, Takahashi K, Sawada K (2017). CO_2_ sensing characteristics of a La_2_O_3_/SnO_2_ stacked structure with micromachined hotplates. Sensors.

[54] Sumangala TP (2018). Effect of synthesis method and morphology on the enhanced CO_2_ sensing properties of magnesium ferrite MgFe2O4. Ceram. Int..

[55] Wang Y, Chyu MK, Wang Q-M (2014). Passive wireless surface acoustic wave CO_2_ sensor with carbon nanotube nanocomposite as an interface layer. Sens. Act. A Phys..

[56] Radhakrishnan JK, Kumara M (2021). Effect of temperature modulation, on the gas sensing characteristics of ZnO nanostructures, for gases O_2_, CO and CO_2_. Sens. Int..

[57] Smith AD (2017). Graphene-based CO_2_ sensing and its cross-sensitivity with humidity. RSC Adv..

[58] Kumar JRN (2022). Polyethyleneimine-chromium oxide nanocomposite sensor with patterned copper clad as a substrate for CO_2_ detection. J. Electron. Mater..

[59] Shinde PV (2020). Room-temperature synthesis and CO_2_ -gas sensitivity of bismuth oxide nanosensors. RSC Adv..

